# Efficacy of Cetylpyridinium Chloride Mouthwash on Denture Plaque Reduction and Microbiome Alteration in a Randomized Crossover Trial

**DOI:** 10.7759/cureus.75357

**Published:** 2024-12-09

**Authors:** Hiroko Tahara, Minoru Sanda, Momoe Itsumi, Haruka Fukamachi, Hiromi Nishi, Fuminori Iwasa, Hirotaka Kuwata, Kazuyoshi Baba

**Affiliations:** 1 Department of Prosthodontics, Graduate School of Dentistry, Showa University, Tokyo, JPN; 2 Department of Oral Microbiology and Immunology, Graduate School of Dentistry, Showa University, Tokyo, JPN; 3 Department of General Dentistry, Hiroshima University Hospital, Hiroshima, JPN; 4 Division of Fixed Prosthodontics, Department of Restorative and Biomaterials Sciences, Meikai University School of Dentistry, Saitama, JPN

**Keywords:** cetylpyridinium chloride, denture plaque, homeostasis, microbiome, oral hygiene

## Abstract

Purpose

This study aimed to evaluate the effectiveness of cetylpyridinium chloride (CPC) mouthwash in reducing denture plaque and its impact on the microbial composition of denture plaque.

Materials and methods

A randomized, placebo-controlled crossover trial included 29 participants with maxillary complete dentures. Participants used either CPC or a placebo mouthwash for one week each in a crossover design. The denture plaque area was quantified using image analysis, and microbiome composition was analyzed via 16S rRNA gene sequencing.

Results

The use of CPC mouthwash significantly reduced the denture plaque area compared to placebo (p<0.025). Microbiome analysis revealed a significant decrease in the relative abundance of the genus *Actinomyces* (p=0.025) and a significant increase in the genus *Haemophilus* (p<0.001) after CPC use. While alpha diversity showed no significant changes, beta diversity analysis indicated a significant shift in microbial composition (p=0.002).

Conclusion

CPC mouthwash effectively reduces denture plaque accumulation and modifies its microbial composition. These findings highlight the potential of CPC mouthwash as an effective tool in denture hygiene management, which may help lower the risk of denture-related oral and systemic infections.

## Introduction

With the increase in the older population, particularly in developed countries, a growing demand for prosthodontic treatment, including removable dentures, is anticipated to restore missing teeth [1]. Acrylic resin, chemically identified as polymethyl methacrylate (PMMA), is the standard material used for removable denture bases due to its affordability, esthetic qualities, and ease of repair. However, this material can act as a scaffold for oral bacteria, referred to as “denture plaque,” because of its inherent porosity [2-4].

Denture plaque is a biofilm that adheres to the denture surface, consisting of microorganisms, their metabolic products, and proteins, such as those from the saliva and serum. Denture plaque can lead to oral conditions, such as halitosis, dental caries, periodontal disease, and denture stomatitis, as well as life-threatening systemic diseases, such as aspiration pneumonia [5-7]. Therefore, daily hygiene management of removable dentures is essential for maintaining both oral and general health in denture wearers. While mechanical brushing is the primary method for cleaning dentures, chemical cleaning with denture cleansers or mouthwashes also plays an important role, especially for older adults with reduced dexterity [8,9].

Cetylpyridinium chloride (CPC) is a cationic ammonium compound with surfactant and bactericidal properties that is commonly used in toothpaste and mouthwashes [10,11]. CPC exerts bactericidal effects by disrupting bacterial cell membranes due to its strong ionic properties [12]. Its surfactant characteristics aid in plaque removal at the gingival margin and inhibit plaque formation within the oral cavity [13,14]. However, the potential role of CPC in controlling denture plaque remains unexplored.

In this study, our primary aim was to evaluate the inhibitory effect of CPC mouthwash on the denture plaque area in patients wearing maxillary complete dentures. The secondary objective was to assess the impact of CPC on the microbial composition of denture plaque. The null hypothesis for this study was that the adhesion of denture plaque following rinsing with CPC solution would be equivalent to that observed after rinsing with a placebo solution.

## Materials and methods

Participants

Maxillary edentulous participants were recruited between April 2021 and December 2023 from the Prosthodontic Clinic of Showa University Dental Hospital. Inclusion criteria required participants to be in adequate general health for dental treatment, have a maxillary complete denture made of acrylic resin, and exhibit no significant clinical problems related to denture use. Exclusion criteria included debilitating systemic diseases; pathological changes, such as candidiasis; poor fit or occlusion upon clinical examination; and dentures relined or repaired with autopolymerizing resin. A sample size calculation indicated a minimum of 14 participants per group, based on an effect size of 0.8, a statistical power of 0.8, and a significance level of 0.05. To account for potential dropouts, 16 participants were recruited per group, resulting in a total of 31 participants (15 male and 16 female; mean age 76.0±9.3 years). All participants provided written informed consent.

Manufacture of CPC and placebo mouthwash solutions

The 0.05% CPC mouthwash was industrially synthesized, while the placebo, used as a control, contained only water and flavoring agents. Both solutions were supplied by Earth Corporation (Tokyo, Japan).

Experimental protocol

On the first day, the maxillary complete dentures were meticulously cleaned using denture brushes, ultrasonic cleaners, and denture cleaning agents (Labalak D; Sundental, Tokyo, Japan) to eliminate denture plaque, which was visually confirmed.

The participants were randomly assigned to Groups A and B using a random number table (FI) according to the design of a randomized controlled trial following a single-blinded assessment. This study was reported in accordance with the Consolidated Standards of Reporting Trials (CONSORT) guidelines. In Group A, the participants were instructed to use the placebo mouthwash solution during the first week (seven days), followed by the CPC mouthwash solution during the second week (seven days), for a total duration of 14 consecutive days. In Group B, the reverse order was followed: The participants used the mouthwash three times daily for 20 seconds after each meal, followed by denture cleaning. Denture cleaning was limited to mechanical brushing of the intaglio and polished surfaces under running water with a denture brush, with the use of denture cleaning agents suspended during the evaluation period. According to the manufacturer’s information, no carryover effects were anticipated for the CPC mouthwash; therefore, a washout period was not incorporated between the evaluation weeks.

All samples were collected by a single investigator (HT) to ensure consistency in data accuracy.

Quantitative denture plaque evaluation

The denture plaque quantity was assessed using a previously published method [15,16]. Following each mouthwash use for one week, the dentures were stained with 0.25% methylene blue dye (FUJIFILM Wako Pure Chemical, Tokyo, Japan), and pictures were taken from the intaglio surface using a white light system (Light Box S; Santec, Tokyo, Japan) and a digital camera (Canon EOS Kiss M2; Canon, Tokyo, Japan). From the images, percentages of the denture plaque area compared to the total denture area were calculated based on pixel counts in the software (Adobe Photoshop vCS6; Adobe, CA, USA).

Bacterial sample collection from denture plaque

To collect bacteria from the denture plaque in group A following the use of the placebo and CPC mouthwash, the stained dentures were immersed in 50 mL of saline solution and subjected to ultrasonic cleaning for 15 min to detach the plaque from the dentures. Then, the separated denture plaque was centrifuged at 3,500 rpm for 5 min (Himac CT6E; Hitachi, Tokyo, Japan). The resulting bacterial samples were stored at −80°C for further analysis.

DNA sequencing and metagenome analysis

Denture plaque samples were analyzed using 16S rRNA amplicon sequencing (Hokkaido System Science, Sapporo, Hokkaido, Japan). Bacterial DNA was extracted using the Extrap Soil DNA Kit Plus ver.2, and the V1-V2 region of the bacterial 16S rRNA gene was amplified using the primer sets 27Fmod and 338R. The base sequences of the DNA fragments amplified from the V1-V2 region were determined using next-generation sequencing (MiSeq; Illumina, CA, USA). The data were processed using the QIIME2 pipeline (v2019.4.0), and operational taxonomic units obtained through clustering were compared to the Greengenes 16S rRNA gene database. The resulting bacterial gene data were deposited with the accession number PRJDB18736.

Bacterial culture and susceptibility test for CPC

*Streptococcus mitis* JCM12971, *S. anginosus* ATCC9895, *Actinomyces naeslundii* JCM8349, and *A. viscosus* JCM8353, *Haemophilus influenzae* ATCC33533 and *H. parainfluenzae* ATCC9796 were grown in Brain Heart Infusion broth supplemented with hemin (10 μg mL-1) and nicotinamide adenine dinucleotide 10 μg mL-1) or Accurate™ Chocolate Agar EXⅡ (Shimadzu Diagnostics, Tokyo, Japan) with the Anaero Pack-CO2 system (Mitsubishi Gas Chemical, Tokyo, Japan) at 37°C. The bacterial susceptibility test for CPC was conducted following the method described by Mohammad et al. [17]. Briefly, 1 mL of bacterial culture was centrifuged, and the resulting pellet was re-suspended in an equal volume of the test solution (placebo or CPC mouthwash). The suspension was shaken and incubated at room temperature for 20 seconds. The reaction mixture was immediately neutralized by adding 10 times its volume of Soybean-Casein Digest Broth with Lecithin & Polysorbate 80 medium (FUJIFILM Wako Pure Chemical). After serial dilution with phosphate-buffered saline, the samples were spread on agar plates, and the number of viable bacteria was determined. The inhibition rate was calculated using the following formula: inhibition rate={(Control CFU−Treated CFU)/Control CFU}×100%.

Statistical analysis

The normal distribution of each dataset was validated using the Shapiro-Wilk test. To evaluate the denture plaque area, for normally distributed data, the unpaired t-test was used to determine potential carryover, treatment, and period effects. In cases where a carryover effect was detected, comparisons were made between the first periods of Groups A and B using the unpaired t-test and between the first and second periods of Group A using the paired t-test. In such instances, the significance level was adjusted to α=0.025 via Bonferroni correction.

As genus-level and individual bacterial flora analysis did not follow a normal distribution, the Wilcoxon signed-rank test was applied, with a significance level of α=0.05. For alpha diversity analysis (Chao1 Index and Shannon Index), which demonstrated a normal distribution, the paired t-test was performed, also with a significance level of α=0.05. Beta diversity was analyzed using the permutational analysis of variance (PERMANOVA) pairwise method. The paired t-test was conducted to determine CPC susceptibility. All statistical analyses were performed using GraphPad Prism, version 5.00 (GraphPad Software, San Diego, CA, USA).

## Results

Participants

**Figure 1:**
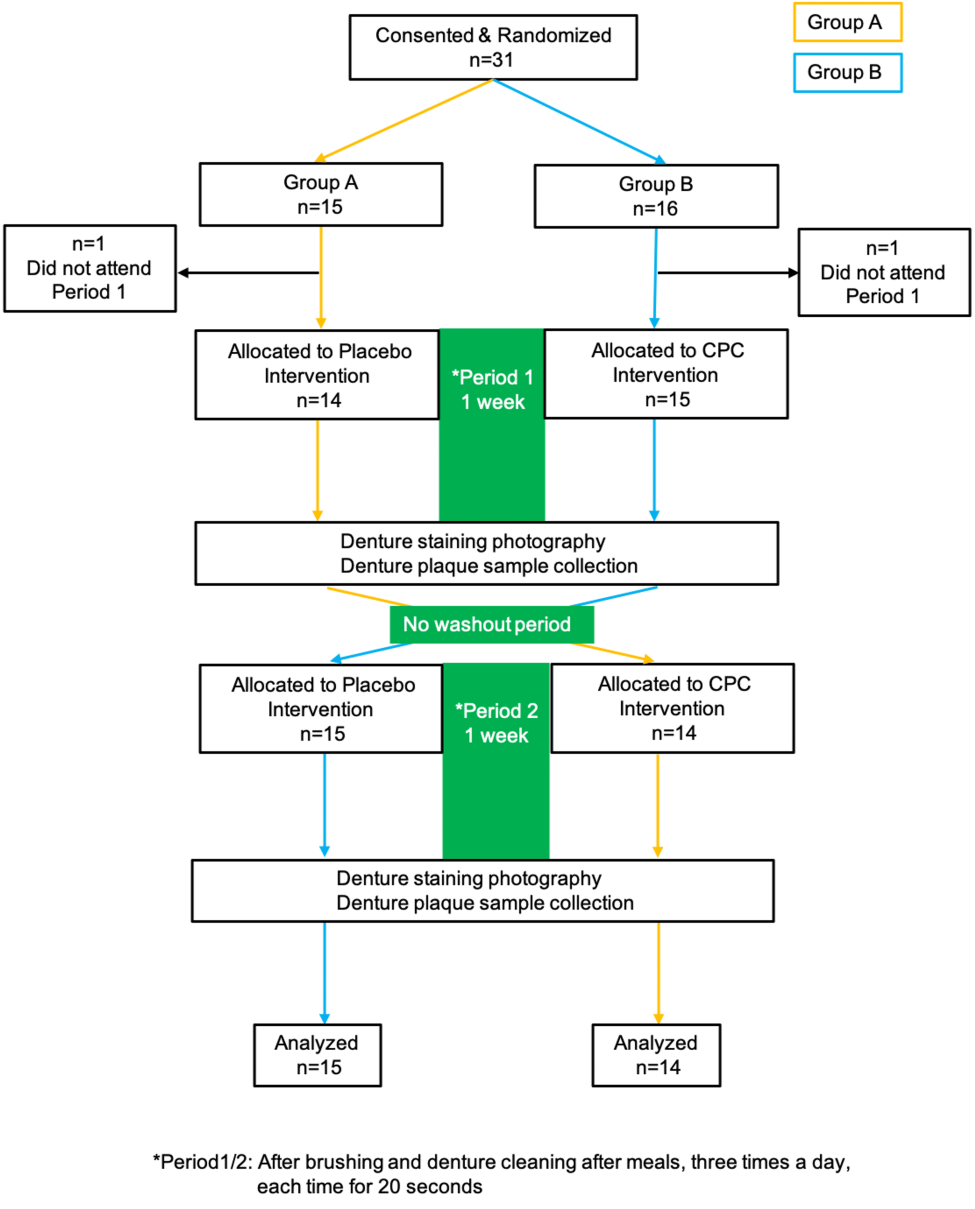
CONSORT flow diagram Participant flow diagram for a randomized crossover clinical trial evaluating the efficacy of CPC mouthwash on denture plaque removal. It illustrates the progression of participants through the study phases, including initial screening, randomization, intervention periods, and final analysis. CPC: Cetylpyridinium chloride; CONSORT: Consolidated standards of reporting trials

Of the 31 participants enrolled (15 male, 16 female participants; mean age±standard deviation: 76.0±9.3 years), as listed with demographic data in Table 1, two were unable to complete the full study protocol due to scheduling conflicts. Consequently, data analysis was performed on the remaining 29 participants (14 male, 15 female; mean age 75.8±9.6 years) (Figure 1).

**Table 1:** The average age and sex of each group at baseline The table presents demographic characteristics of the study participants, including sex, age, and denture usage duration. Age is expressed as mean±standard deviation. Data include both participants who completed the study and those who dropped out.

	Group A	Group B
Male sex, n	6	9
Female sex, n	9	7
Age (years)	73.2±10.2	78.6±7.6
Denture usage duration (years)	1.6±2.1	2.5±2.6

Statistical analysis of crossover study

The washout period between the first and second weeks was omitted because of the assumption that there was no carryover effect based on the manufacturer’s information. However, statistical analysis showed a carryover effect of the CPC mouthwash (p=0.036, Table 2).

**Table 2:** Evaluation of carryover effects Analysis of carryover, treatment, and period effects in the crossover study. Statistical analyses were performed using unpaired t-tests with a significance level of α=0.1. The carryover effect indicated a significant residual effect when cetylpyridinium chloride (CPC) was used in the first period. The treatment effect shows a significant difference between CPC and placebo treatments. * p<0.1; *** p<0.001.

Test	Estimate	95% confidence interval	p-value
Carryover effect	30.1	2.16–58.0	0.036*
Treatment effect	11.5	6.48–16.6	<0.001***
Period effect	0.455	−4.61–5.52	0.855

Image analysis of denture plaque area

**Figure 2:**
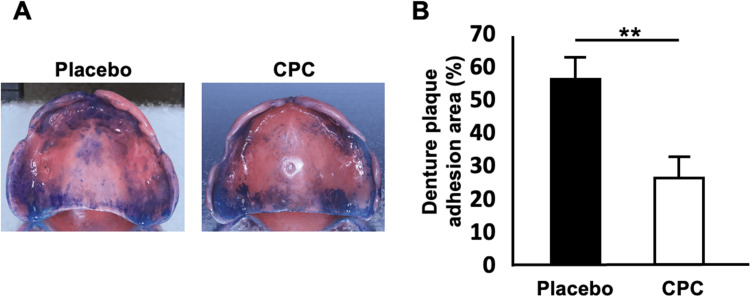
Comparison of denture plaque adhesion area between placebo and CPC groups in the first period of the study A: Representative photographs of the maxillary complete dentures stained with methylene blue. Left: Placebo mouthwash treatment (Group A, period 1). Right: CPC mouthwash (Group B, period 1). B: Quantitative analysis of denture plaque adhesion area in Group A (n=14) and Group B (n=15) after the first period. Bar graph illustrating the mean percentage of the denture plaque adhesion area relative to the total intaglio surface of dentures. Error bars represent standard deviation. Statistical analysis was performed using the unpaired t-test with a significance level of α=0.025. ** p<0.025. CPC: cetylpyridinium chloride

**Figure 3:**
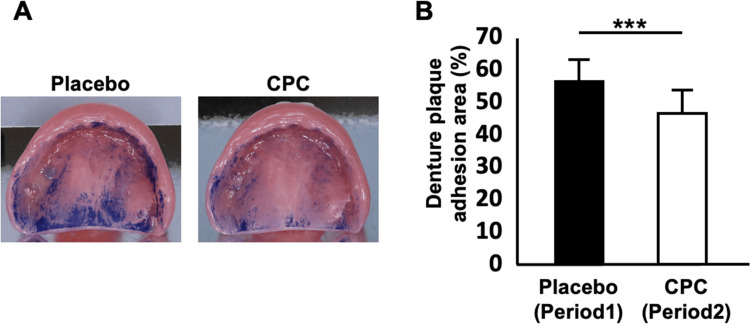
Comparison of denture plaque adhesion area between placebo and CPC mouthwash treatments in Group A across treatment periods A: Representative photographs of maxillary complete dentures stained with methylene blue. Left: Placebo mouthwash treatment (Group A, period 1). Right: CPC mouthwash treatment (Group A, period 2). B: Quantitative analysis of denture plaque adhesion area in Group A across treatment periods (n=14). Bar graph illustrating the mean percentage of the denture plaque adhesion area relative to the total intaglio surface of dentures. Error bars represent standard deviation. Statistical analysis was performed using a paired t-test with a significance level of α=0.025. *** p<0.001. CPC: cetylpyridinium chloride

In the first week, a comparison between Groups A and B revealed that Group B, which received the CPC mouthwash intervention, exhibited a significantly smaller denture plaque area compared to Group A, which used the placebo mouthwash (Group A: 55.6±5.1%; 95% confidence interval (CI): 44.6-66.5, n=14; Group B: 29.0±6.0%; 95% CI: 14.4-41.9, n=15; p=0.002) (Figure 2). A longitudinal analysis within Group A showed a significant reduction in the denture plaque area during the second week, when CPC mouthwash was used, compared to the first week, when the placebo mouthwash was used (first week with placebo: 55.6±5.1%; second week with CPC: 44.5±5.0%; 95% CI: 33.8-55.2; n=14; p<0.001) (Figure 3).

Evaluation of the denture plaque bacterial flora

**Figure 4:**
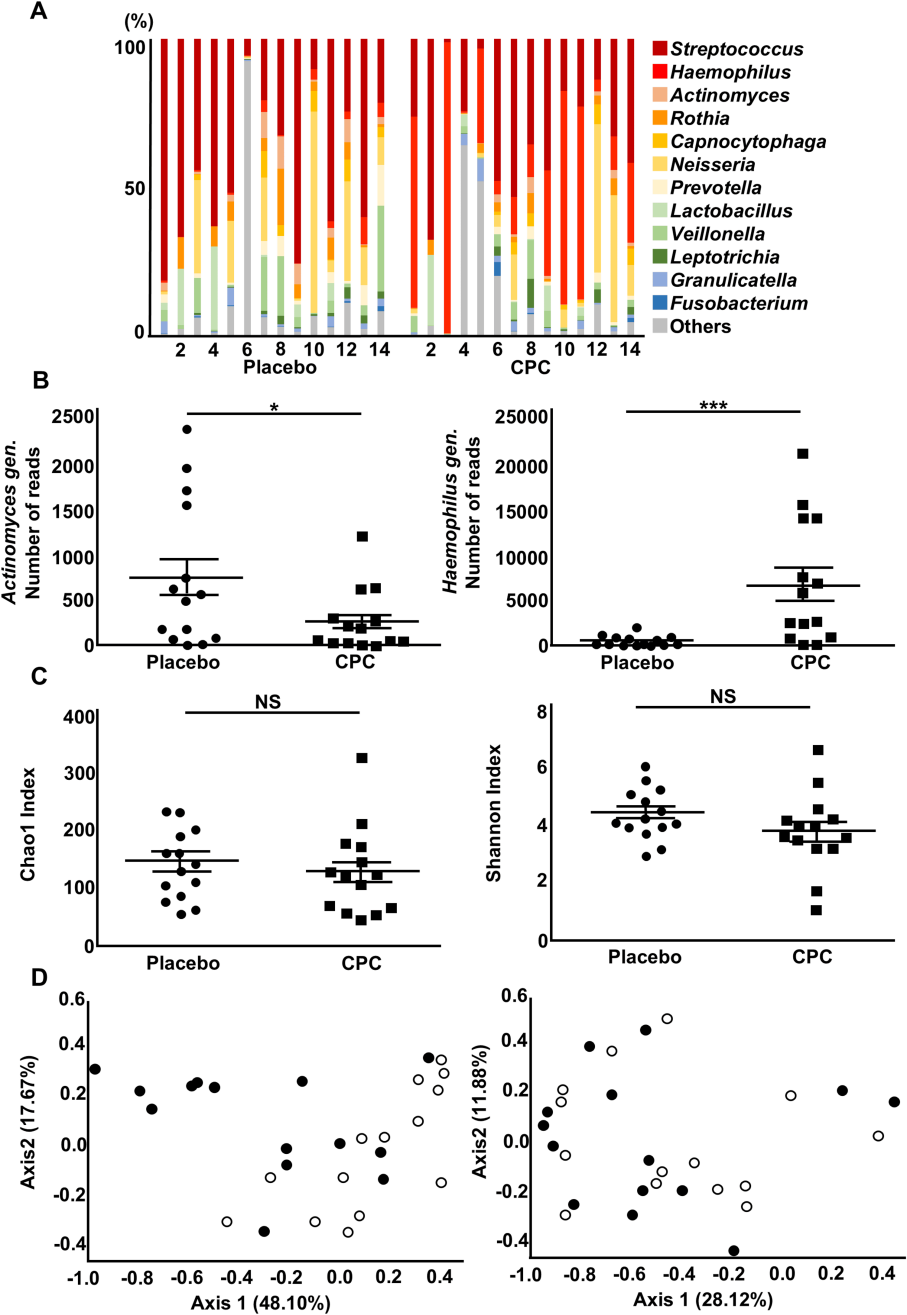
Microbiological analysis with next-generation sequencing A. Bacterial flora analysis of Group A using next-generation sequencing Bar charts comparing analysis of the genus-level microbial composition of denture plaque samples from Group A (n=14) after intervention with the placebo and CPC mouthwash solutions. The vertical axis shows the relative proportion of reads for each bacterium. The numbers in the horizontal axis correspond to participants, while different colors indicate different bacterial genera. The diversity of the bacterial flora was confirmed for each participant. B. Comparison of specific bacteria with significant differences in their relative abundance The chart compares the number of bacterial sequence reads for *Actinomyces* (left) and *Haemophilus* (right) genera after mouthwashing. Each dot represents a sample, with the horizontal lines indicating the mean and standard errors. For genus-level bacterial community analysis, the Wilcoxon signed-rank test was applied at a significance level of α=0.05. * p<0.05; *** p<0.001. C. Comparison of alpha diversity between the placebo and CPC groups Graphs show the Chao1 (left) and Shannon (right) indices. Each dot represents a sample, with the horizontal lines indicating the mean and standard errors. For alpha diversity analysis (Chao1 Index and Shannon Index), the paired t-test was performed at a significance level of α=0.05. NS: not significant. D. Comparison of beta diversity between the placebo and CPC groups Principal coordinate analysis plots of microbial community profiles in denture plaques. Beta diversity was compared between placebo (black circle) and CPC (white circle) groups using the pairwise permutational analysis of variance (PERMANOVA). A significant difference was found in the weighted UniFrac distance (left, p<0.05), but not in the unweighted UniFrac distance (right). CPC: cetylpyridinium chloride

The bacterial flora in Group A, without any carryover effects, was assessed using next-generation sequencing (Figure 4A). A total of 107 genera were identified. Among these, the genus *Actinomyces* showed a significant decrease in relative abundance (p=0.025), while *Haemophilus* exhibited a significant increase (p<0.001) (Figure 4B). Species-level analysis revealed a reduction in the abundance of *Haemophilus influenzae*, *Haemophilus parainfluenzae*, *Streptococcus infantis*, and *Streptococcus anginosus* following CPC exposure.

Furthermore, we analyzed alpha diversity, which evaluates species diversity and its distribution and uniformity in the sample. The Chao1 index, representing species diversity, showed no significant variation between the placebo and CPC treatments (Chao1 Index, p=0.606) (Figure 4C). Similarly, the Shannon index, representing species diversity and population distribution, exhibited no notable differences (Shannon Index, p=0.166) (Figure 4C).

Effect of CPC on the growth of each bacterium

To investigate the effect of the CPC mouthwash solution on each bacterium’s growth, we assessed the growth of *S. infantis*, *S. mitis*, and *S. anginosus*. The same verification was also carried out using *A. naeslundii*, *A. viscosus*, *H. influenza*, and *H. parainfluenzae*. The effect on growth was examined by culturing each bacterium in a medium containing CPC mouthwash solution. The results are presented in Table 3.

**Table 3:** Sensitivity of bacteria to CPC mouthwash solutions The bacterial species identified through metagenomic analysis—*Streptococcus anginosus*, *Actinomyces naeslundii*, *A. viscosus*, *Haemophilus influenzae*, and *H. parainfluenzae*—were cultured on BHI agar with either CPC or placebo mouthwash solutions. All bacteria inhibit the growth treated with CPC. Growth inhibition was assessed by counting bacterial colonies. Bacterial counts were compared between the placebo and CPC groups using paired t-tests with a significance level of α = 0.05. ***p < 0.001 indicates a significant reduction compared to the placebo. CPC: cetylpyridinium chloride.

	S. mitis	S. anginosus	A. naeslundii	A. viscosus	H. influenzae	H. parainfluenzae
Placebo mouthwash solution (CFU/mL)	3.42×10^8^	4.00×10^9^	6.93×10^8^	1.57×10^9^	1.56×10^９^	2.07×10^10^
CPC mouthwash solution (CFU/mL)	1.44×10^6^	5.30×10^5^	5.33×10^7^	9.9×10^4^	2.49×10^6^	8.83×10^7^
Growth inhibition ratio (%)	99.6***	100***	92.3***	100***	99.8***	99.6***

## Discussion

Previously, studies have demonstrated the effectiveness of mouthwashes in reducing plaque adhesion on natural tooth surfaces and the tongue [13,14]. This aligns with the findings of our study, further supporting the potential of mouthwash in promoting oral hygiene. However, to our knowledge, this study is the first to clinically evaluate the effectiveness of mouthwashes containing CPC on inhibiting denture plaque. 

In this study, a significant reduction in denture plaque area was observed in both cross-sectional comparisons between participants and longitudinal comparisons within the same participants when transitioning from a placebo mouthwash solution to CPC. Consequently, the null hypothesis was rejected.

Because of its high hydrophobicity and lack of electrostatic repulsion, PMMA allows bacteria and organic components in saliva to adhere to it. In addition, PMMA becomes porous due to polymerization shrinkage, and its surface becomes roughened at the microscopic level during the polishing process during cleaning, which enhances denture plaque adhesion and accumulation [18,19]. To counteract these properties, it is important to implement effective hygiene measures to maintain clean dentures.

We have shown that denture plaque adhesion can be inhibited by coating the denture surface with methacryloyloxyethyl phosphorylcholine (MPC) polymer [15]. However, MPC is a coating material specifically designed for dentures and can only exert its effects on them. In contrast, mouthwashes containing CPC can act not only on dentures but also on remaining natural teeth, suggesting the potential usefulness of CPC.

In this study, a next-generation sequencer was used to analyze the bacterial flora in denture plaque before and after using CPC. Next-generation sequencers can analyze microbial flora accurately without the need for culturing [20,21] and are gaining recognition as a method for comprehensively analyzing the spectrum of the bacterial species in the oral cavity. This technology is also applicable to the analysis of denture plaque as examined in this study. Denture plaque biofilm on dentures interacts with oral microbes and is related to systemic diseases, such as aspiration pneumonia and denture stomatitis [22,23]. Next-generation sequencing enables the analysis of various bacteria, including unculturable obligate anaerobe bacteria [24], allowing for a quantitative assessment of how denture materials and cleaning methods affect oral flora. This study evaluated CPC rinse effects from a microbiological perspective.

NGS analysis revealed significant changes in the abundance of *Actinomyces* and *Haemophilus genera*. *Actinomyces*, a gram-positive anaerobic or facultatively anaerobic bacterium commonly found in the oral cavity, contributes to the formation of periodontal pockets and dental plaques [25]. *Actinomyces* species are known to increase in the deep regions of periodontal pockets and are involved in the progression and onset of gingivitis and periodontal disease [26]. In this study, the significant reduction of *Actinomyces* species following the use of CPC mouthwash suggests that CPC may prevent infections caused by *Actinomyces* species, such as periodontal disease. *Haemophilus*, a gram-negative, facultatively anaerobic, rod-shaped bacterium, is distributed throughout aerobic and anaerobic oral environments and exerts pathogenicity in the condition of immunocompromised hosts [27]. The genus *Haemophilus* has been reported to increase in gingivitis patients treated with CPC [14]. However, the relationship between the degree of gingivitis and the increase of *Haemophilus* remains unclear. Our study also demonstrated that CPC treatments promoted an increase in *Haemophilus* within denture plaque. These suggest that the increase in *Haemophilus* may serve as a potential indicator of oral health. CPC treatment increased the proportion of *Haemophilus* species; however, culture experiments revealed more potent growth inhibition of *H. influenzae* and *H. parainfluenzae* than *S. mitis*. Further investigation is required to resolve this discrepancy. Recent findings have indicated that the spatial arrangement of oral bacteria affects their metabolic interactions and antimicrobial resistance [28]. Therefore, evaluating the effects of CPC requires the assessment of both the individual species and the overall bacterial community.

Alpha diversity showed no significant change before and after CPC mouthwash use. However, beta diversity, analyzed using weighted UniFrac distance, showed a significant change. CPC may selectively affect specific bacterial groups, thereby potentially suppressing certain pathogenic or dominant bacteria. Further species-level research is required to explore its long-term effects on oral microbial ecosystems.

This study was originally designed as a crossover trial without a washout period because the manufacturer indicated no carryover effect was expected from this mouthwash, aiming to shorten the study period and prevent participants from dropping out. However, a carryover effect was observed with the CPC mouthwash solution. This prevented the use of statistical methods, such as analysis of variance (ANOVA), which are normally used in crossover studies. Therefore, the evaluation of denture plaque adhesion was conducted through a cross-sectional comparison between Groups A and B during the first period and a longitudinal comparison within Group A between the first and second weeks. The results showed a statistically significant reduction in dental plaque with the CPC mouthwash compared to the placebo group (p=0.0023, p=0.0003). This indicates that the absence of a washout period did not compromise the purpose of this study.

In addition, the results of the carryover effect analysis indicated that continuous use of the CPC solution mouthwash for one week would persist for at least the following week. The effect persisted after discontinuation of the CPC solution, uncovering unexpected findings.

A limitation of this study is that only bacterial 16S rRNA sequences were analyzed, and the effects on fungi were not considered. The oral environment is an ecosystem where bacteria and fungi coexist; a comprehensive analysis that includes fungi, such as *Candida*, is required to discuss the reduction in the risk of various complications related to dental plaque. Further investigations encompassing a broader spectrum of microbial flora, including fungal species, are warranted.

## Conclusions

The evidence suggests that rinsing with a CPC mouthwash three times a day for approximately 20 seconds for one week effectively reduces denture plaque adhering to the mucosal surface of maxillary complete dentures. Although it causes minimal significant changes in the types of bacteria compromising the bacterial flora, CPC’s inhibitory effect on the growth of certain bacterial species indicates that it may influence the composition of the denture plaque biofilm. Therefore, CPC mouthwash represents an effective strategy for managing denture hygiene and mitigating the risk of denture-related oral and systemic infections.
